# Knowledge of and adherence to standard precautions in a hemodialysis unit: a cross-sectional study

**DOI:** 10.1590/1516-3180.2021.0288.23072021

**Published:** 2022-02-23

**Authors:** Laura Prado Medeiros, Kamila Silva de Miranda, Thayna Martins Gonçalves, Deusdélia Dias Magalhães Rodrigues, Karen Renata Nakamura Hiraki, Marilia Duarte Valim, Monica Taminato, Richarlisson Borges de Morais

**Affiliations:** I Nurse Resident, Multiprofessional Residency Program, Universidade Federal de Uberlândia, Uberlândia (MG), Brazil.; II Nursing Student, Universidade Federal de Uberlândia, Uberlândia (MG), Brazil.; III Nursing Student, Universidade Federal de Uberlândia, Uberlândia (MG), Brazil.; IV MSc. Nurse, Hospital de Clínicas, Universidade Federal de Uberlândia, Uberlândia (MG), Brazil.; V PhD. Associate Professor, Instituto de Ciências Biomédicas, Universidade Federal de Uberlândia, Uberlândia (MG), Brazil.; VI PhD. Adjunct Professor, Faculdade de Enfermagem, Universidade Federal de Mato Grosso, Cuiabá (MT), Brazil.; VII PhD. Adjunct Professor, Escola Paulista de Enfermagem, Universidade Federal de São Paulo, São Paulo (SP), Brazil.; VIII MSc. Doctoral Student and Assistant Professor, Escola Técnica de Saúde, Universidade Federal de Uberlândia, Uberlândia (MG), Brazil.

**Keywords:** Universal precautions, Accidents, occupational, Patient safety, Nursing, team, Renal dialysis, Standard precautions, Nursing staff, Worker’s health, Occupational health, Patient safety, Hemodialysis

## Abstract

**BACKGROUND:**

Standard precautions (SPs) are recommended safety measures for healthcare professionals to follow, with a view to preventing healthcare-related infections (HCRIs) and for their own protection. Inadequate adherence to these measures can lead to occurrences of occupational accidents and HCRIs.

**OBJECTIVES:**

To ascertain the knowledge of and adherence to SP measures among the nursing staff of a hemodialysis service and the relationship of these variables to occurrences of work accidents with biological material.

**DESIGN AND SETTING:**

Descriptive cross-sectional and correlational study with a quantitative approach developed in a hemodialysis clinic in Minas Gerais.

**METHODS:**

Data were collected through sociodemographic questionnaires and questionnaires on knowledge of and adherence to SPs.

**RESULTS:**

29 professionals participated in the study. It is noteworthy that all of them had already participated in training related to SPs. However, no relationship was identified between knowledge of (15.17 points) and adherence to (71.86 points) SPs. In addition, inferential analysis showed that there was a relationship between suffering a work accident with biological material and the sociodemographic data and knowledge of and adherence to standard precautions.

**CONCLUSION:**

Knowledge of the SPs that had been established did not mean mastery of the subject. Despite positive results regarding adherence, factors requiring improvement were observed. It was possible to infer the characteristics that gave rise to greater risk of occurrences of accidents at work. Thus, this study showed the importance of assessing knowledge of and adherence to SP, in order to optimize and direct continuing education towards resolving occupational exposure.

## INTRODUCTION

According to the Centers for Disease Control and Prevention (CDC), standard precautions (SPs) are essential safety measures for healthcare with a view to protecting the client against healthcare-related infections (HCRIs), and for professional protection against occupational exposure to potentially contaminated biological material (PCBM).^1^

This set of SP measures is composed of the following: hand hygiene before and after contact with patients or areas adjacent to them; use of personal protective equipment (PPE), including glasses, mask, apron and procedure gloves; correct handling and disposal of sharps; and vaccination against hepatitis B.^2,3^

It is known that low adherence to SPs contributes to growing numbers of occupational accidents in healthcare services.^3^ Professionals who do not make adequate use of PPE and who handle sharps are exposed to occupational risks. In this context, the nursing professionals of hemodialysis services can be highlighted, given that they handle sharp devices and potentially contaminated equipment on a daily basis.^4,5^

It is important to consider that chronic renal patients who are undergoing renal replacement therapy (RRT) are in a vulnerable condition. They are subjected to invasive procedures every week, which are performed in a hospital environment, where pathogens and multidrug-resistant agents are present.^6^ Studies have demonstrated occurrences of infection among patients on RRT,^7,8^ thus highlighting the prevalence of infections caused by resistant microorganisms among patients on dialysis and awaiting kidney transplantation.^9^

In view of the complexity of the hemodialysis service and the vulnerability of patients subject to RRT, the nursing team at these services takes on an important role of responsibility for care, in order to ensure patient safety and their own safety through adherence to SP.

However, studies have revealed low adherence to SP among nursing professionals^10,11^ and have identified factors that determine inadequate adherence. Recklessness, shortage of materials in hospital units,^12^ insufficient knowledge and work overload have been highlighted.^13^

In view of the above, we perceived that there was a gap in the literature. In particular, there seemed to be a need to assess the knowledge of and adherence to SP among the nursing team working on hemodialysis, considering that these variables are directly related to occurrences of HCRIs, occupational accidents with exposure to PCBM and the quality and safety of healthcare.

## OBJECTIVE

The aim of this study was to ascertain the knowledge and adherence of the nursing staff of a hemodialysis unit with regard to SP measures and correlate these variables with occurrences of occupational accidents with PCBM.

## METHODS

This study followed the recommendations of the Strengthening the Reporting of Observational Studies in Epidemiology (STROBE) initiative.^14^ It was a descriptive quantitative cross-sectional and correlational study performed at a hemodialysis clinic in the city of Uberlândia, Minas Gerais, Brazil. This is a private clinic accredited to the Brazilian National Health System (Sistema Unico de Saúde, SUS) that performs an average of 3,500 hemodialysis sessions per month.

A non-probabilistic or convenience sample was used. All the nursing professionals at the institution who had worked there for more than six months with a minimum workload of 20 hours a week were eligible for inclusion in the study, provided that they were not on vacation, maternity leave or sick leave at the time of data collection.

Professionals whose work exclusively involved administrative activities were excluded. In addition, those who were participating in training related to safety measures at the time of data collection were also excluded, as this training would possibly overvalue their knowledge about SP.

### Data collection and statistical analysis

Data were collected using self-administered questionnaires: one seeking sociodemographic data and variables relating to occurrences of occupational accidents with PCBM; a questionnaire to assess knowledge of SP (Standard Precautions Knowledge Questionnaire, SPKQ);^15^ and another questionnaire to measure adherence to SPs (Standard Precautions Adherence Questionnaire, SPAQ).^16^

The SPKQ had previously been validated for Brazilian realities. It contains 20 questions relating to healthcare professionals’ knowledge of SP, and has proved to be stable due to its reliability according to the intraclass correlation coefficient, which was calculated as 0.91, and its satisfactory agreement with the mean kappa index.^15^

The SPAQ is another instrument that has been translated and validated for use in Brazil.^16^ It is composed of 20 questions relating to adherence to SP, in a Likert-type format, ranging from 1 to 4 points for each question.

For data compilation, double entry was used independently, in order to eliminate possible mistakes. The data were analyzed by means of the R software, version 3.6.3, and the R Studio software, version 1.2.5001 (Integrated Development for R; RStudio, PBC, Boston, MA, United States).

Descriptive statistical techniques were used for presentation of the numerical data. Categorical data were expressed in terms of frequencies (absolute and relative). To identify the relationship between occurrences of accidents with PCBM and the other variables studied, bivariate analysis was performed using Fisher’s exact test. Simple and multiple linear regression models were used to identify the relationship of the scores obtained in the SPAQ and the SPKQ with occurrences of accidents with PCBM. The significance level was taken to be alpha < 0.05.

### Ethical issues

This study was approved by our institution’s ethics committee for research on human beings, under CAAE number 09987318.7.0000.5152, dated July 17, 2019. Confidentiality and anonymity for the participants was ensured, in accordance with resolution no. 466/2012 of the National Health Council.

## RESULTS

Twenty-nine nursing professionals participated in the study who were active in the outpatient dialysis service of our institution participated in this study. These comprised 24 nursing technicians (82.75%) and five nurses (17.24%); 79.31% were female; the participants’ mean age was 39.1 years; their mean length of professional experience was 13.0 years; and their mean length of experience of dialysis was 11.9 years (Table 1).

**Table 1.** Distribution of the nursing staff according to gender, age, education level, accidents with sharps, accidents with potentially contaminated biological material, participation in training, vaccination status against hepatitis B and knowledge of vaccine response, at a hemodialysis clinic in the city of Uberlândia, Minas Gerais, Brazil, 2020 (n = 29)

Variables	n	%
**Participants’ gender**		
Male	6	20.69
Female	23	79.31
**Age (years)**		
20 to 30	5	17.24
30 to 40	10	34.48
40 to 50	10	34.48
50 to 60	2	6.89
> 60	1	3.44
**Education level reached**		
Completed high school	19	65.51
Undergraduate at university/college	4	13.79
Graduated from university/college	1	3.44
Specialization	5	17.24
**Work accidents with PCBM**		
No	16	55.17
Yes	13	44.82
**Accidents with PCBM consisting of unhealthy skin or mucus, involving contact with potentially contaminated blood or body fluids**		
No	19	65.51
Yes	10	34.48
**How many times have you had an accident with PCBM consisting of unhealthy skin or mucus, involving contact with potentially contaminated body fluids?**		
None	18	62.06
Once	3	10.34
Twice	2	6.89
Four times	2	6.89
Several (more than four)	3	10.34
Missing response	1	3.44
**Accidents at work with biological material by means of sharps or exposure to mucous membranes or unhealthy skin in the last 12 months (last year)**		
No	24	82.75
Yes	5	17.24
**Participation in training on standard precautions offered by the employing institution**		
Yes	29	100.00
**Schedule for complete hepatitis B vaccination (3 doses)**		
Yes	29	100.00
**Underwent a medical examination to detect antibodies for hepatitis B**		
No	1	3.44
Yes	28	96.55
**Examination result regarding antibodies for hepatitis B**		
Positive	14	48.27
Negative	8	27.58
I don’t know	3	10.34
Missing response	4	13.79

n = absolute number; % = percentage; PCBM = potentially contaminated biological material.

Regarding participation in training relating to SPs, 29 (100%) responded that they had had some training: 26 (89.65%) underwent this in 2019 and the majority (55.17%) received their training from the institution’s Internal Accident Prevention Commission (IAPC). It was noteworthy that 26 (89.65%) of the nursing staff surveyed expressed a desire to receive training and updates on SP measures.

When asked about changing devices for disposing of sharps when they reached two-thirds of their capacity, 21 (72.41%) reported that the device was changed when it reached its maximum recommended capacity, while eight (27.58%) reported that they did not do this replacement.

In analyzing the responses to the SPKQ, the average knowledge of SPs was 15.17 points; the maximum score was 18 and the minimum was 5 points. With regard to adherence to SPs (SPAQ), the mean score was 71.86 points, the maximum was 80 and the minimum was 45 points. The relationship between knowledge of and adherence to PP was calculated by means of Kendall’s correlation between the scores. A weak correlation of 0.221 (P = 0.126) was noted, which showed that there was no relationship between knowledge of and adherence to SPs in the sample studied.

The majority of the participants correctly judged each item in the SPKQ questionnaire. It was noteworthy that 79.31% said that they knew what SPs were. However, 82.75% considered question 3 (Q3) to be true, which states that SPs have the main objective of protecting the healthcare team, thereby devaluing patient safety. In addition, more than 41% of the professionals surveyed judged Q18 to be incorrect or did not know how to judge Q18: “When providing nursing care to patients with hepatitis C or human immunodeficiency virus (HIV), it is necessary to adopt only the SP measures” (Table 2).

**Table 2.** Descriptive analysis on knowledge of standard precautions in a nursing team at a hemodialysis clinic in the city of Uberlândia, Minas Gerais, Brazil, 2020 (N = 29)

Knowledge of standard precautions	True/yes	False/no	I don’t know
Q1 - Do you know what standard precaution measures are?	23 (79.31)	0 (0.0)	5 (17.24)
Q2 – Standard precautions should only be applied to patients diagnosed with infection or patients who are in the incubation period for a given infection.	4 (13.79)	20 (68.96)	3 (10.34)
Q3 - The main objective of adhering to standard precaution measures is to protect the healthcare team.	24 (82.75)	2 (6.89)	3 (10.34)
Q5 - Hand hygiene should be performed when providing care to different patients.	26 (89.65)	2 (6.89)	0 (0)
Q16 - Symptomatic respiratory patients (coughing, sneezing, etc.) must be kept at least one meter away from other patients in the ward.	22 (75.86)	2 (6.89)	4 (13.79)

Regarding adherence to SP, it was highlighted that 6.89% of the nursing professionals performed hand hygiene before providing assistance to patients only rarely or sometimes. In procedures with the possibility of contact with urine or feces, 100% of the participants reported always wearing gloves. However, in procedures with the possibility of contact with unhealthy skin, adherence to use of this equipment decreased (82.75%). Regarding the use of goggles and a mask, 79.31% stated that they always used this equipment. However, only 48.27% use the respiratory etiquette when coughing or sneezing (Table 3).

**Table 3.** Descriptive analysis on adherence to standard precautions in a nursing team at a hemodialysis clinic in the city of Uberlândia, Minas Gerais, Brazil, 2020 (n = 29)

Adherence to standard precautions	N	R	S	O	A
Q1 - I perform hand hygiene before assisting the patient.	0 (0)	1 (3.44)	1 (3.44)	7 (24.13)	20 (68.96)
Q14 - I use a protective mask when there is the possibility of contact with splashes of blood, body fluids, secretions or excretions.	0 (0)	0 (0)	1 (3.44)	3 (10.34)	23 (79.31)
Q15 - I wear goggles when there is a possibility of contact with splashes of blood, body fluids, secretions or excretions.	0 (0)	1 (3.44)	4 (13.79)	1 (3.44)	23 (79.31)
Q18 - I do not recap used needles.	5 (17.24)	4 (13.79)	3 (10.34)	5 (17.24)	10 (34.48)
Q20 - When coughing or sneezing, I use a disposable handkerchief to cover my mouth and nose, and then I dispose of it and sanitize my hands.	1 (3.44)	2 (6.89)	7 (24.13)	5 (17.24)	14 (48.27)

N = never; R = rarely; S = sometimes; O = often; A = always.

In correlating occurrences of biological accidents with PCBM and other variables, significance at the 5% level was identified through Fisher’s exact test. There were associations with the following response variables: suffering an occupational accident with PCBM (P < 0.001); suffering a work accident with PCBM through needlestick or exposure to mucous membranes or unhealthy skin over the last 12 months (P = 0.036); the frequency with which occupational accidents with PCBM were notified (P <0.001); results from examinations for antibodies to hepatitis B post-vaccination (P = 0.021); responses to question 1 (Q1) of the SPKQ, “Do you know what the standard precautions (SP) measures are?” (P = 0.002); responses to Q6 of the SPKQ, “Since the use of gloves can prevent hand contamination, it is not necessary to clean your hands after removing the gloves” (P < 0.001); responses to Q18 of the SPKQ, “When providing nursing care to patients with hepatitis C or HIV, it is necessary to adopt only the standard precaution measures” (P = 0.004); responses to Q20 of the SPAQ, “When I cough or sneeze, I use a disposable handkerchief to cover my mouth and nose, then I dispose of it and clean my hands” (P = 0.049).

Univariate analysis was carried out on responses to the indicator of suffering an accident with PCBM. All the variables of the SPKQ and SPAQ were used as covariates. Variables with significance at 10% in univariate analysis were then subjected to multivariate analysis.

In making multivariate adjustments using the significant variables, the variables in the final multivariate model that explained suffering an accident with PCBM with a significance level of 5% were the following: occurrence of a work accident with potentially contaminated sharps (standard deviation, SD: ± 1.409; confidence interval, CI: [0.935; 6,867]; P = 0.019); and identifying question 18 of the SPKQ as false, i.e. “When providing nursing care to patients with hepatitis C or HIV, it is necessary to adopt only standard precaution measures” (SD: ± 1.297; CI: [0.566; 6.054]; P = 0.028).

Thus, the odds ratio (OR) between occurrence of work accidents with potentially contaminated sharps and the variable “If you suffered a work accident with potentially contaminated sharps”, showed that nursing professionals who reported having already suffered this type of accident were 27 times more likely to have accidents in this way than were those who had not suffered a work accident with sharps (OR: 27.51; CI: [2.546; 959.731]; P = 0.019). In addition, those who believed that the sentence in question SPKQ-18 was false (“When providing nursing care to patients with hepatitis C or HIV, it is necessary to adopt only standard precaution measures”) were 17 times more likely to suffer an accident through working with PCBM than were those who believed that the sentence was true or did not know whether they believed the sentence (OR: 17.27; CI: [1.761; 425.760]; P = 0.028).

## DISCUSSION

After recruiting nursing professionals and applying the questionnaires, the sociodemographic description and the relationship between occurrences of occupational accidents and other variables studied were assessed.

There was a predominance of professionals at technical level, which can be explained by the provisions of Ordinance No. 389/14, which determines that in hemodialysis services, one nursing technician is required for every four patients and one nurse per shift is required for every 35 patients.^17^ Thus, it is inferred that the demand for professionals at technical level will always be greater than the demand for nurses.

In a previous study, it was observed that 61.7% of the population of nursing professionals was up to 40 years old.^18^ This corroborates our results, in which the average age was found to be 39.1 years. The prevalence of young adult professionals explains the average of 13 years of professional experience.

Another result presented by 100% (29) of the professionals was that they had all participated in training provided by the employing institution within the last 12 months, with a SP approach. Their responses to Q1 of the SPKQ (“Do you know what standard precautions measures are?”) may have been determined by this fact, given the impact of continuing education in healthcare services, with a view to expanding knowledge and preparing the team for work activities,^2,12,19^ especially those with active methodology and protagonism of the subject.^15^ However, even so, it was observed that 17.24% of the professionals stated that they did not have any knowledge about SPs.

Although 79.31% of the sample claimed to know about SPs, the antagonistic result stood out, in which 82.75% responded that Q3 of the SPKQ was true (“Adherence to standard precaution measures has the main objective of protecting the healthcare team”), which incorrectly presents the main objective of SPs. A similar result was observed in a study in which 67.5% of the nurses believed that the main objective of SPs was to protect the healthcare team, while ignoring the protection provided for patients that was involved in adherence to SPs.^12^

On the other hand, a study carried out in São Paulo showed results that were contrary to those of previous studies, in which 87.6% of the participating nurses disagreed with the statement contained in Q3 of the SPKQ.^15^ This result was also described in another recent study, in which 72% of the participants strongly disagreed with the objective of the SPs that is presented in that question.^20^

The participants in our study showed positive results regarding the SPAQ questions that investigate the frequency of hand hygiene at different times or situations, namely: Q1 - before providing assistance to the patient; Q2 - before performing aseptic techniques; and Q3 - after exposure to potentially contaminated body fluids. This result demonstrates the coherence and ability of the professionals to recognize the moments when hand hygiene is essential. However, even though these results were satisfactory, it is important to highlight that for Q1 of the SPAQ, 6.89% of the participants presented an incoherent response to the recommendations. On the other hand, this was not repeated in Q3 of the same instrument. This suggests that nursing professionals are concerned with sanitizing their hands in order to minimize the risk of contamination when exposing themselves to potentially contaminated fluids, but give less value to the importance of this act before providing nursing care, which thus represents an important risk to patients’ safety.^21^

From a study developed in a hemodialysis unit, it was pointed out that nursing professionals had low adherence to hand hygiene, and that they performed this technique only in 16.6% out of 1090 opportunities.^22^ Although hand hygiene is frequently discussed, educational interventions remain one of the important alternatives for raising professionals’ awareness of this.^23,24^

There was significant divergence in the responses to the assertion in Q18 of the SPKQ (“When providing nursing care to patients with hepatitis C or HIV, it is necessary to adopt only the standard precaution measures”). Among the professionals surveyed, 38% judged that this assertion was incorrect and 3% did not know how to judge it. This was in contrast to their self-reported knowledge of SP and drew attention to the fact that the hemodialysis unit studied serves patients who are seropositive for HIV and hepatitis B and C. This result was similarly observed in two other studies: in one, only 85% judged the same statement to be correct;^12^ while in another study, 76.8% marked this statement as true.^15^ In addition, this finding highlights the existence of stigma in caring for patients who are known to be positive for these viruses. This attitude puts nursing professionals in a situation of greater occupational risk, due to their unnecessary (excessive) and inappropriate use of PPE.

The relationship between occurrences of accidents with PCBM and the SPKQ variables shows the impact of knowledge of SPs on the prevalence of occupational accidents, since accidents with PCBM are usually associated with nonuse or inappropriate use of PPE and non-adherence to SPs.^25,26^ This can be shown by the result in which 80% of the professionals who suffered accidents with PCBM did not believing that just adopting SPs was enough for care directed to patients with hepatitis C or HIV.

The risk of accidents with potentially contaminated sharps was 27.51 times greater among professionals who had already had an accident. This corroborated a result observed in another study, in which 46.6% of the sample reported having already been exposed to PCBM and 63.5% of these individuals claimed to have been injured more than once.^26^

The importance of training in terms of biosafety practices can be emphasized: not only at work, but also in professional training, as a strategy for minimizing the impacts of lack of knowledge,^27^ since occupational accidents with PCBM may be associated with lack of knowledge or low participation in training activities.^26^ In addition, our findings contribute to directing institutional managers’ attention towards professionals who are at greater risk of suffering new accidents at work with PCBM. Our findings emphasize training measures for promoting a better institutional safety climate and, thus, for also promoting health safety (for both patients and professionals) in the institution.

Although the average scores obtained from the questionnaires (SPAQ = 15.17 and SPKQ = 89.82) were positive, the deficits in knowledge of and adherence to SPs become significant in view of the vulnerability of users of the renal replacement therapy service and the possible consequences for workers, such as occupational accidents and their associated morbidity, in addition to possible losses for the institution.^3^

In another study, it was concluded that knowledge of SPs did not necessarily mean adherence to these measures.^12^ This corroborates what was observed in our sample, since only a weak correlation was identified between these two variables.

It is worth mentioning that the present study was carried out in a single center, which limited the population for composition of the sample. It can therefore be suggested that further studies should be conducted, with participation of several dialysis services, in order to confirm and generalize the findings from the present study, for nursing professionals working in these services.

## CONCLUSION

Although the institution evaluated in this study had provided training on SPs within the last 12 months, the professionals surveyed showed significant inconsistencies in their knowledge of SPs. We can highlight their inappropriate perception of insecurity regarding use of SPs to care for people with viral conditions such as HIV and hepatitis C.

Regarding adherence to SP, although the overall result was positive, there were reports of non-adherence to hand hygiene before performing nursing care, insufficient adherence to respiratory etiquette and inadequate needle recapping. This occurrences demonstrate that there is a need for improvements in adherence to these measures among the nursing professionals surveyed.

Furthermore, this study highlights the importance of training healthcare teams to provide them with better knowledge of and adherence to SP. It provides a stimulus for undertaking other studies in order to expand the understanding of this problem among healthcare professionals who are exposed to potentially contaminated biological material in their work practice, especially with regard to professionals working in dialysis services, given their daily exposure.

Lastly, the relationship between occurrences of accidents with potentially contaminated sharps and lack of knowledge of SPs corroborates data from the literature. This highlights the importance of evidence-based practice in order to optimize health safety, both for professionals and for patients.
